# Supplementary material for: Mutations in PCYT2 disrupt etherlipid biosynthesis and cause a complex hereditary spastic paraplegia
Source: Brain. 2019 Oct 22;142(11):3382–97. doi: 10.1093/brain/awz291 (PMC6821184; doi:10.1093/brain/awz291)
Supplement: awz291_Supplementary_Data [file awz291_supplementary_data.zip › awz291-Suppl_data/Supplementary_Data4.zip!/Supplementary information 3 - fibroblast lipidomics.zip!/Fibroblast lipidomics/Controle vs Patient1/Statistical plots and data/Volcano plot.pdf]

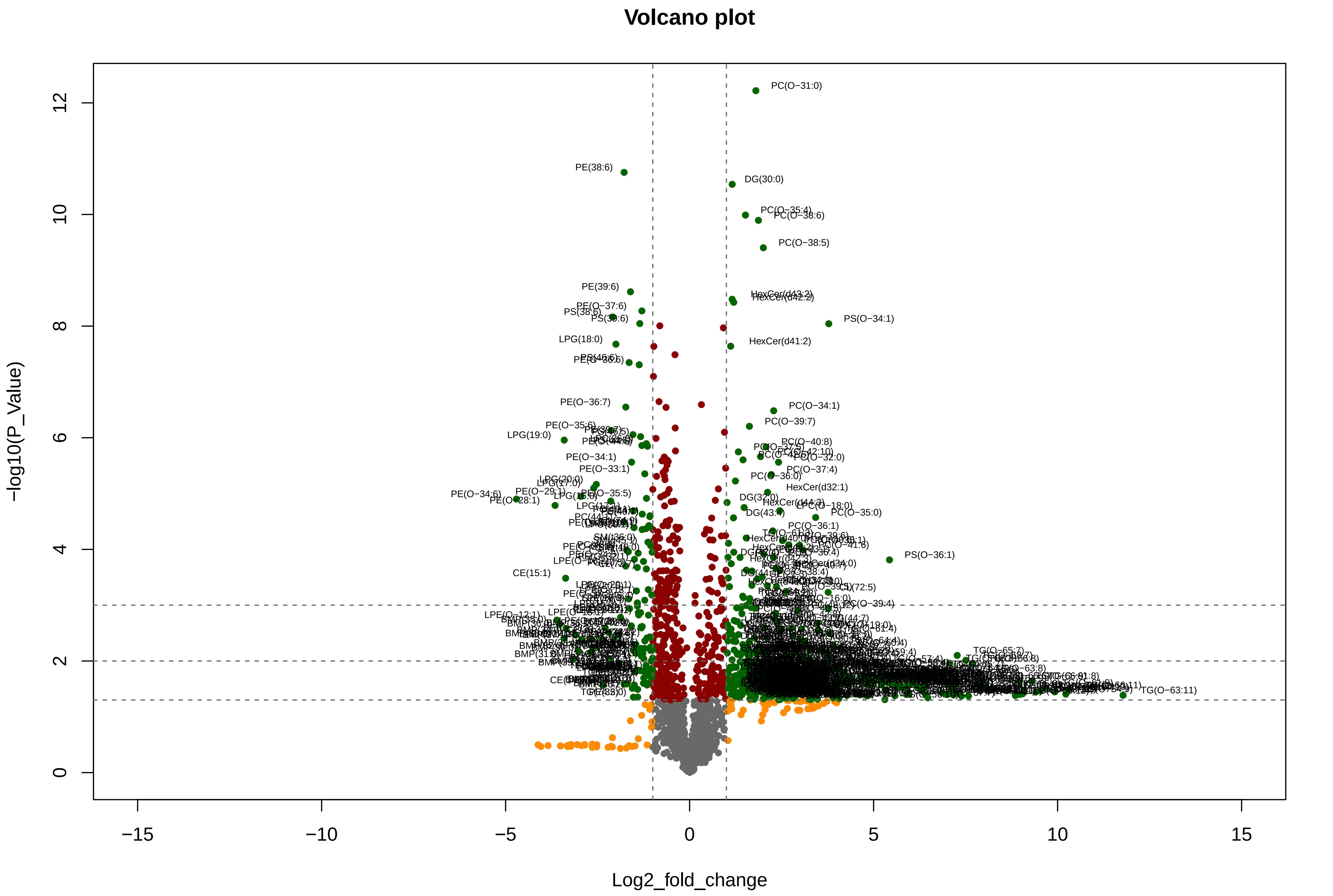
Volcano plot
-log10(P_Value)
Log2_fold_change
0
2
4
6
8
10
12
−15
−10
−5
0
5
10
15
PC(O−31:0)
PE(38:6)
DG(30:0)
PC(O−38:6)
PC(O−38:5)
PE(39:6)
PS(O−34:1)
HexCer(d41:2)
LPG(18:0)
PE(O−36:7)
PC(O−34:1)
PC(O−39:7)
LPG(19:0)
PE(O−34:1)
PE(O−33:1)
PE(O−34:6)
PE(O−28:1)
HexCer(d32:1)
PC(O−35:0)
PC(O−36:1)
PS(O−36:1)
CE(15:1)
TG(O−63:11)
